# SoCap YMH – youth mental health, social capital and help-seeking: a study protocol

**DOI:** 10.3389/fpubh.2024.1406649

**Published:** 2024-06-11

**Authors:** Mikael G. Ahlborg, Antony Morgan, Petra Svedberg, Jens M. Nygren, Malin Eriksson, Katrin Häggström Westberg

**Affiliations:** ^1^School of Health and Welfare, Halmstad University, Halmstad, Sweden; ^2^Department of Public Health, Glasgow Caledonian University in London, London, United Kingdom; ^3^Department of Social Work, Umeå University, Umeå, Sweden

**Keywords:** adolescence, co-production, help-seeking, mental health, social capital

## Abstract

**Background:**

The increase in adolescents reporting mental health problems presents a major public health challenge. The complex association between mental health and social capital motivates further investigation of social capital as a crucial aspect in shaping adolescents’ help-seeking knowledge, attitudes, and behaviours.

**Aim:**

This protocol presents a project that aims to investigate social capital in relation to help-seeking and mental health in close collaboration with adolescents and key stakeholders in the school setting, in the southern part of Sweden.

**Methods:**

A mixed-method design with three interconnected work packages (WP) will be undertaken with an emphasis on co-production where adolescents are involved throughout the process. WP1 is a development and validation of two questionnaire instruments for assessing social capital and help-seeking in adolescence. WP2 is a longitudinal quantitative study involving 1,500 adolescents from two regions representing rural and suburban/urban settings. Adolescents aged 15 will be asked to complete questionnaires concerning social capital, mental health, and help-seeking in a baseline and one-year follow-up, allowing for investigation of the role of social capital for help-seeking. WP3 is designed to elucidate experiences and knowledge of adolescents and key stakeholders via collaborative World Café workshops. These will be held along the project to evolve the generated knowledge and maximize it’s applicability during and after the project is finalized.

**Conclusion:**

The results are expected to further the understanding of the relationship between adolescents’ social capital, mental health, and help-seeking, to contribute to a deeper understanding of the mechanisms behind the paradoxical help-seeking patterns among adolescents today and to narrow the gap between research and practice to produce sustainable and efficient strategies, which may facilitate help-seeking and improve the mental health of adolescents within existing organizational structures.

## 1 Introduction

### 1.1 Adolescent mental health

The surge in mental health problems among adolescents globally presents a pressing societal concern (1–5). Sweden is not exempt from this development (6). For example, over recent years there has seen a substantial rise in the number of contacts made to the health care system by adolescents reporting an array of mental health problems (7). This is substantiated by an accompanying rise in drug prescriptions for depression and anxiety. Despite these increasing rates, it is pertinent to note that a significant number of adolescents refrain from seeking support or have long help-seeking trajectories (8, 9). In broad terms, paradoxically, adolescents who suffer from more severe mental health problems may be less inclined to seek help (10) and the increase in healthcare utilization, which is seen in Sweden, is mainly driven by adolescents with less severe forms of mental health problems (11).

This development has prompted researchers to seek deeper insights into the factors influencing adolescents’ mental health and help-seeking behaviours. In this endeavour, there is a need for a more nuanced approach to mental health assessment and reporting than is presently the case (12). One reason for this pertains to the increased reporting of mental health problems among adolescents, which dilutes the previous benefits of using threshold values to determine what level of mental health problems should raise concern. There is an urgent need to develop strategies and tools to better distinguish between normal responses to the stressors of life that adolescents naturally face during their development years and those mental health problems that require professional support and treatment. One factor of interest that continues to soar in mental health research is social capital (13, 14). The concept of social capital connects to the social dimension of mental health and refers to the sum of resources that reside within social networks (15) that its members contribute to, benefit from and are affected by.

### 1.2 Social capital and mental health

Resources embedded in social networks have been identified as important for the mental health of adolescents (16). It is well known that social network involvement provides various forms of social support that could decrease stress and thus benefit mental health outcomes (17, 18). Further, social networks could benefit mental health by providing opportunities to learn new skills and by giving a sense of belonging and meaningfulness. In addition, involvement in social networks could influence mental health and help-seeking through the influence of peers as role models for behaviour (19). However, social capital comes in different forms, which might have different effects on health. Theorists, such as Bourdieu, Coleman and Putnam, put social capital on the map of social sciences during the early 90s. Although protagonists of social capital, they take different disciplinary perspectives. While Bourdieu (15) emphasizes social hierarchies, mutual recognition and personal profit as preservers of social reproduction in society, Coleman (20) and Putnam (21) share a more optimistic view of social capital that relies upon reciprocity and democratic engagement. For adolescents, Bourdieu’s view interprets into social structures based on social status, behaviour and norms, as well as preconceptions and expectations from both peers and adults. The other perspective can be exemplified through mutual and unconditional support and trust within a friend network or within the family, enjoy spending time with like-minded and feeling safe within a context or group, building on a reciprocal and positive view of people in general. It is important to consider these differing perspectives when conceptualizing social capital for adolescents, while being responsive to the implications of the transformation of adolescents’ social arenas, seen over the past decade. Regardless of which perspective seems more appealing, the commonalities as we see it lie within the importance of social networks “for getting on and getting ahead in life” (22).

The concept has a cognitive and a structural dimension, consisting of bonding, bridging and linking ties that manifest through norms, behaviours, and attitudes (23). *Bonding* social capital consists of strong ties among people in homogenous networks, which can strengthen common identities and function as a source of help and support among the network members (24). *Bridging* social capital, instead consists of weaker ties among people in heterogeneous networks, which can serve as an important source of information and resources (21, 25). Linking social capital in the adult population often refers to inter-hierarchical ties, but this has not been given much attention with reference to adolescents. Instead, a broader notion of bridging social capital has been suggested to include the hierarchical ties of linking social capital (22).

Evidently, social capital is a broad and multidimensional concept. Overall, adolescents have been less included in the conceptualization of the concept (26), suggesting that social capital has generally been considered a biproduct of childhood that may benefit them in the future rather than a useful asset during childhood (27). The “asset approach” to social capital acknowledges adolescents as active agents in shaping their social networks (14) and recent research highlights the importance of incorporating this view for better understanding of the link between social capital and adolescent mental health (28).

The connection between social capital and mental health is complex and previous attempts to establish a clear causal link where a decline in social capital precedes poor mental health have provided limited evidence (29, 30). This is somewhat expected given the multidimensionality of social capital and its embeddedness into the social dimension of mental health, in addition to the multifactorial origins of mental health problems and the interplay of hereditary factors in mental illness incidence (31).

There are resources within adolescents’ social relationships and networks that appear universally desirable. Both theoretical underpinnings (22) and empirical research (16) indicate that trust, sense of belonging, connectedness, reciprocity, and support help shape the mental health of adolescents as they transition into adulthood. Adolescents themselves describe that by having access to safe spaces, to feel connected to networks that nurture sociability, and maintaining control in social interactions are important aspects of these resources (28). These components emphasize the importance of fostering supportive relationships and cultivating a sense of belonging for adolescents.

In the intricate landscape of studying the interplay between social capital and mental health among adolescents, a challenge lies in the translation between theoretical conceptualization and a valid operationalization of the concept in measurement. Existing research highlights the complexities and current limitations in capturing the multidimensional aspects of social capital that resonate uniquely with adolescents (26). There appears to be a dearth of instruments that effectively encapsulate the constructs of social capital and the breadth of relevant social contexts specific to adolescents underscores the necessity for methodological advancements. While previous studies have succeeded in translating social capital theory into measurement techniques for certain constructs of social capital (32, 33), a more inductive and comprehensive approach may be required to align measurement strategies with the dynamics of adolescents’ social networks of today. Another challenge lies in the transferability of measurement between different social cultures, both within and between nations and groups. What resources are accessible and most prominent may differentiate depending on social norms, perhaps especially in relation to mental health. This challenging endeavour promises to pave the way for a nuanced understanding of how social capital influences mental health, ultimately informing targeted interventions that bridge the gap between theoretical concepts and practical applications for reversing the current trend of adolescent mental health. Fitting previous qualitative work into the frames of social capital theory is a challenging task. Many existing instruments for assessment of social capital have been developed with a top-down approach, grounded in theory and with adolescents as passive recipients. However, a strictly inductive approach to the conceptualization of social capital for adolescents may infer flaws pertaining to content validity. An abductive approach, i.e., an oscillation between existing theory and adolescents’ perceptions and experiences, may therefore provide upsides that both sets the initial boundaries for social capital, but also allows for unique insights within these defined boundaries (34).

### 1.3 Social capital, mental health and help-seeking

An intriguing aspect of the association between social capital and mental health is adolescents’ propensity to seek help. Adolescents grappling with severe mental health problems may be less inclined to reach out for help compared to peers experiencing milder issues that occur naturally during adolescence (11). As adolescents navigate their social environments, the connections they establish and the networks they engage with might serve as crucial determinants shaping their attitudes and behaviours towards seeking help. These relational structures and the resources that reside within them could foster an environment, encouraging of open discussions about mental health problems that nurtures competency in help-seeking. Consequently, adolescents with higher levels of social capital might find seeking help a more viable and normative course of action, thereby enhancing their readiness to access appropriate support, both non-professional and professional. There is, however, an ongoing discussion about the “dark sides” of social capital, where strong bonding networks also nurture “unhealthy” norms and behaviours (35) that may have a diametrically opposite impact on help-seeking behaviours and, it cannot automatically be assumed that higher social capital equates healthy help-seeking behaviours. In addition, mistrust of public authorities, including health care, can grow within strong bonding social networks (36). On the contrary, Myeong and Seo (36) also show that bridging ties may help strengthen the trust for public authorities. Connected to this, bonding networks might foster norms of “taking care of their own problems” rather than seeking help from professionals and health care. Paired with more severe mental health problems and a lesser inclination to seek help, this poses a delicate problem in need of a thorough investigation in empirical research. Resources such as trust may have different meaning in relation to help-seeking depending on the norms that reside in social networks (37). The cognitive and structural components of social capital may also play different roles. While cognitive dimensions can both limit and foster awareness of mental health issues, structural dimensions enable connections through which adolescents access tangible resources and guidance, especially through bridging social capital (36). In unravelling the nuances of this association, we open up an avenue for valuable insights that could contribute to enhancing mental health care for adolescents.

As for mental health help-seeking within the adolescent group, validated instruments are rarely employed in research. Instead, single-use measures or vignettes tend to be utilized (38) and few instruments that assess help-seeking focus on capturing knowledge on available help (39). Moreover, there seems to be equal interest given to the frequency of measuring attitudes and intentions, as to assessing actual previous help-seeking behaviour (40). Instruments focusing on help-seeking among adolescents commonly concentrate on four facets: attitudes towards seeking help, intentions to seek help, fears related to treatment concerning help-seeking, and barriers to help-seeking (38). A comprehensive help-seeking instrument may lack completeness without encompassing knowledge on available help as well as information on actual help-seeking behaviour. The urgency to develop a comprehensive and updated instrument for understanding mental health help-seeking among adolescents is paramount, especially considering the transformation of help-seeking into a partially digitalized environment.

## 2 Aims and objectives

The overall aim of this project is to investigate social capital in relation to help-seeking and mental health in close collaboration with adolescents and key stakeholders in the southern part of Sweden. The following research questions will be explored in three different work-packages;

1. How can social capital and help-seeking among adolescents in Sweden be assessed with high validity and reliability in relation to mental health?
2. What is the role of social capital for mental health and help-seeking in adolescence?
3. How can the knowledge generated throughout the project be translated into practice?

**Figure 1:**
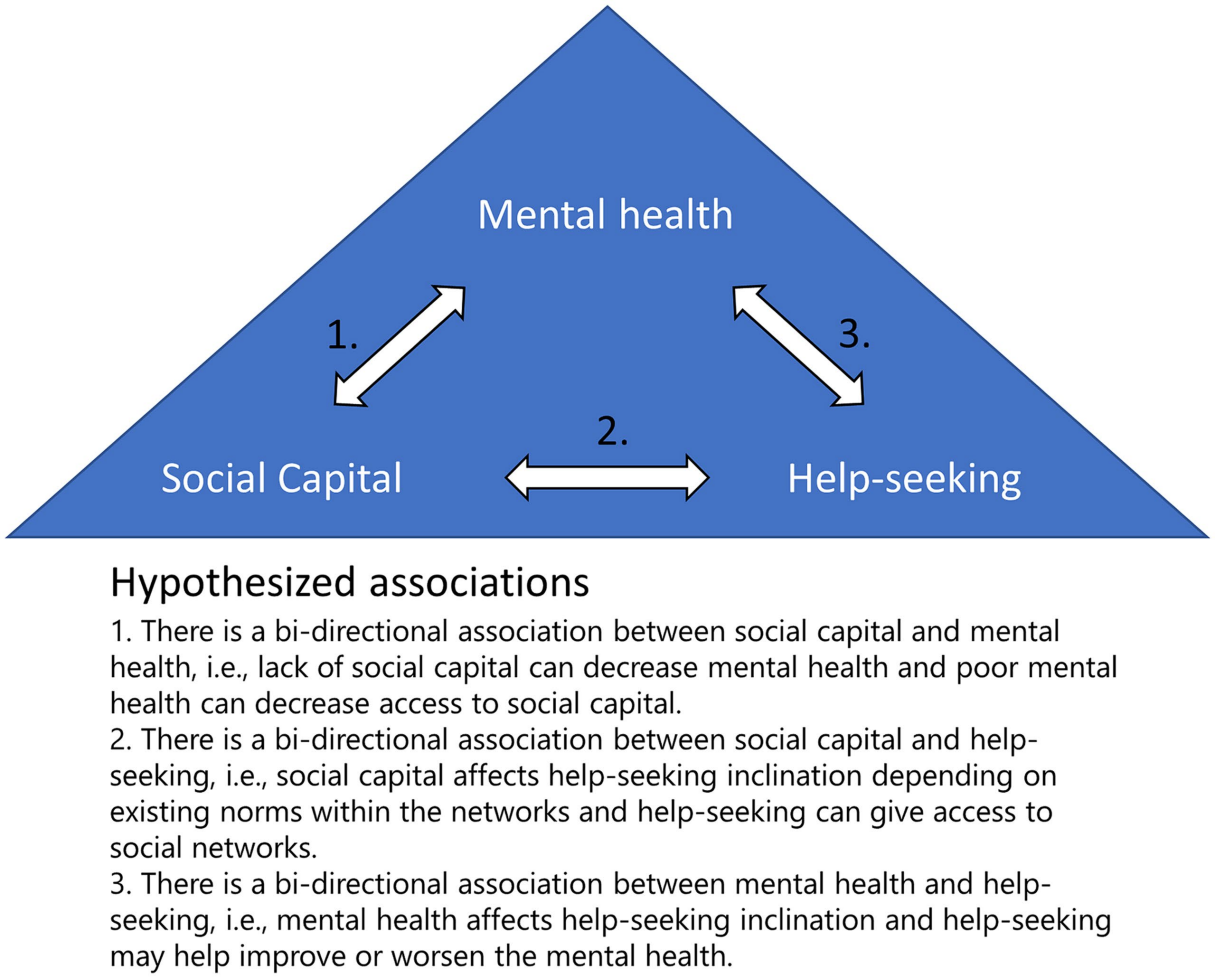
Hypothesized association between mental health, social capital and help-seeking.

The project at hand is designed to disentangle some of the complexities of the bi-directional association between social capital, help-seeking and mental health among adolescents (see Figure 1). Through understanding more about this complex interplay, insights can be gained into how interventions can be strategically employed to identify vulnerable adolescents and facilitate mental health care access among adolescents. In addition, an exploration into how social capital shapes adolescents’ attitudes towards seeking help provides a critical lens through which we can better understand the pathways leading to timely intervention and support the help-seeking process. This aligns with the broader aspiration of ensuring that adolescents, irrespective of the severity of their mental health problems, receive timely and appropriate support during their transition into adulthood.

## 3 Methods and analysis

### 3.1 Study design

This project includes mixed methods, combining a qualitative participatory framework and quantitative longitudinal procedures. It consists of three work packages (WP), and stretches over a 3 year-period, focusing primarily on adolescents in secondary school. The first and third WP uses a participatory research approach, described previously in research by members of our research team (41). This approach includes both consultative and collaborative levels of participation in order to enhance involvement and empowerment of adolescents. To allow for a thorough investigation of social capital, mental health and help-seeking among adolescents, the involvement of adolescents to ensure relevance and improve the quality of research methodology is essential. Key stakeholders, such as representatives from municipalities, social services and schools, will be involved during the entire process to ensure relevance and applicability of research findings. The design and the scientific methods of this project have been developed through dialogues between researchers, health care professionals, representatives from municipalities and a national patient organization.

### 3.2 Setting

The schools are situated in three different districts in southwest Sweden, representing rural and urban settings. Sweden has a population of 10.5 million people (scb.se) and is considered a high-income country with a relatively low Gini Index, although rising rapidly (42). The healthcare system is publicly financed via local taxation and is structured into three levels; local, regional and national (43). Nurse-led school healthcare focuses primarily on preventive care and regular health check-ups, immunization etc. The school healthcare also offers counsellors and occasional visits from general physicians. The number of days per week that the school nurse and counsellor are present at each school can vary significantly, depending on the size of the school and local decision-making. Apart from that, healthcare services available to adolescents consist primarily of youth clinics and primary care. However, this system has been characterized as fragmented (9, 44).

### 3.3 WP 1 – instrument development

This WP will address the first research question, i.e., How can social capital and help-seeking among adolescents in Sweden be assessed with high validity and reliability in relation to mental health? The WP is designed to tackle the absence of validated instruments that comprehensively encapsulate the concepts of both social capital and help-seeking as articulated by adolescents. Its objective is to create two distinct instruments catering to these specific aspects. It is of importance to establish instruments that cover both relevant contexts and provide an image of both the structure of networks and the perceived resources within them. The procedure follows a standardized methodology for developing and validating instruments within social and behavioural health research, put forward by Boateng et al. (45). The methodology provides a step-by-step guide accompanied by explanations of common pitfalls and successful practices. Combined with the participatory approach of the study, this ensures active collaboration with adolescents in (1) item development, (2) instrument development, and (3) evaluation of the instruments (45). The WP extends previous research undertaken by the research team, where a systematic review and evaluation of existing instruments shows a lack of adolescent involvement during development (26) and qualitative field work involving adolescents, which elucidates the importance of safe spaces, connectedness and predictability in adolescents’ social networks (28), and will now also involve adolescents from a suburban/city setting.

#### 3.3.1 Participants and recruitment

Recruitment of adolescents will be made possible by previously established contacts with principals in two different school districts representing rural, and urban setting. Researchers will provide oral and written information to 9th grade classes during school hours. They will also be available to address any queries or concerns raised by the students. Students will be informed about the outline of the project, the specific study and details that adhere to the requirements for informed consent according to the Declaration of Helsinki (46) and the Swedish national ethics authority. Students are eligible for participation when they have turned 15 years of age and do therefore not require consent from parents if they are willing to participate. Students are asked to return signed consent forms to their teachers within 10 days. Researchers will then collect consent forms upon next visit.

#### 3.3.2 Focus groups

Focus-group interviews (47) will be conducted as a first step in item development for both instruments covering social capital as well as help-seeking. The groups will include six to eight pupils per focus-group session. Teachers will be consulted before focus groups are formed. School hours, pupil relationships and gender distribution will be considered in this process. The participatory work will have an inductive approach, aiming to have adolescents describe social capital and help-seeking, respectively, in relation to mental health. This will allow adolescents to share their lived experience of the two phenomena. Focus group discussions with an inductive approach are appropriate to identify dimensions of the concepts of interest (45). A semi-structured interview guide will be formed beforehand, framed within a broad definition of social capital but inspired by previous qualitative research on social capital in relation to adolescent mental health (28). The sessions will be held at the schools, during or directly after school hours, in designated meeting rooms or similar with promise of no disturbance. The estimated time for each session will be 60 min. Each session will start with a short presentation of the researchers and the aim of the study. Then, opening questions will be used to get a general idea of the participants’ understanding of mental health. Next, questions relating to the exploration of social capital will be asked, such as *what people or networks do you consider to be important for the well-being of adolescents in the 9th grade in general?, what is it that makes them important*? and *if you are not feeling well mentally, what is it about people that makes you feel you can confide in them?* Follow up questions will be asked to have adolescents elaborate on their thoughts or exemplify. Creative cards with examples of general networks, network members and social contexts (e.g., school, neighbourhood) will be used as a tool to facilitate discussions initially. Questions on help-seeking will be asked, such as *what do you seek help for in regard to mental health, where do you seek help* and concerning the time-aspect, *when is the appropriate time to seek help* and *what time-perspective is useful for investigating help-seeking among adolescents?* The adolescents will also be asked to reflect on seeking help in digital versus physical environments. Two members of the research team will attend the focus-group interviews, one as a moderator and one to take notes. Sound will be recorded digitally.

#### 3.3.3 Data analysis and item generation

According to Boateng et al. (45), item generation preferably includes a combination of theory, previous empirical research and qualitative data gathering. For this study, the first step will be to transcribe the qualitative data from the focus group interviews verbatim. Then, a Grounded Theory methodology known as Situational Analysis (48) will be applied to the data related to social capital. This methodology will allow for all data to be recognized during item generation, which is an important feature for the following item reduction and face validity procedures (45). Analysis of the data related to help-seeking will follow the same methodology but focus more on processes than situations.

The item generation for both instruments will be conducted jointly by the research team and include a back-and-forth process between the results of the qualitative analysis, theoretical literature, and previous empirical research (inclusive of existing validated instruments). Following recommendations, the initial item pool will be at least twice the size of what a finished instrument may hold and purposefully include items that are tangential or unrelated to the core construct (45). Wording and response options of the items should be unambiguous and designed with care to capture the experiences of the adolescents, while adhering to the standards of content validity and prevention of confirmation, recollection, and statistical bias.

#### 3.3.4 Face and content validity

The next step of developing the two instruments comprises evaluating each of the items for content relevance, representativeness, and quality (45). This will be done through evaluation by experts within the field (content validity) and group sessions with adolescents (face validity). The expertise within the research team on social capital theory and help-seeking will be complemented by independent experts within the field and key professionals working with adolescents locally and nationally to serve as a DELPHI-panel (49) during content validity procedures.

Expert opinion and reviews from adolescents will be used to revise items and improve the understandability of the complete instruments. Apart from a face validity session with adolescents, a second session will focus on design of the two instruments. There are recommendations for questionnaire design regarding response options and type of questions (50) that will be considered, but it is also important to allow for input of the target group during the design phase. Existing social capital instruments that capture the structure of social networks have for example included name generators and mapping of social relationships (51). This session will also explore different ways to assess help-seeking put forward by the adolescents. The two instruments will consequently be digitalized in the Sunet Survey software and made accessible online for pilot testing and WP 2.

#### 3.3.5 Pilot testing and instrument evaluation

The third step is to evaluate the psychometric properties of the two instruments through a pilot study with 9th grade adolescents from each school district (a total sample size of 200–300). This will allow evaluation of construct validity and reliability, including factor analysis, internal consistency, convergent and divergent validity (45). Convergence will be tested by using an existing scale, alternatively blocks of items from existing scales to match with the scales developed in this project. For the social capital scale, these blocks are not predetermined since WP1 has an inductive and participatory development process, and there is no golden standard for the measurement of Social Capital. The Family Affluence Scale (52) will be used as a proxy-measure for socioeconomic status to allow for divergent validity testing. All pupils in the ninth grade at the schools involved in WP1 will be asked to fill out the questionnaire digitally, except for pupils that have participated in focus group interviews and face validity sessions. Participation is voluntary and anonymous, meaning that no personal information or background information except gender will be gathered.

### 3.4 WP 2 – baseline and 1-year follow-up

The main research question addressed in this work package is: What is the role of social capital for mental health and help-seeking in adolescence? The second WP is a longitudinal population study (53) of adolescents in the school-setting, designed to establish a baseline and a first-year follow-up to investigate how social capital is linked to help-seeking in adolescence and how social capital may facilitate early identification of adolescents at risk of developing severe mental health problems in need of treatment and support. It is well known that the prevalence of mental health problems increases during early adolescence (54), however, social capital tends to be more stable during these years apart from a transformation in the structure of social networks, which is mostly related to the expansion of peer networks (55). Therefore, we propose a one-year follow-up will provide valuable information into the changes of, and relationship between, mental health, social capital and help-seeking.

#### 3.4.1 Participants and recruitment

The sample in the second WP extends to another school district and will consist of adolescents from three municipals in the western part of Sweden, from the 9th grade (15–16 years old at baseline), representing different socioeconomic levels, living conditions, community settings and gender. All secondary schools in each district will be asked to participate in the study. In schools eligible for participation, researchers will inform the pupils during school hours about the study, how to access information and consent forms and the content of the questionnaire. Students consent to participate via an online form before accessing the online survey.

#### 3.4.2 Sample size

Sample size, 300–500 adolescents per municipality (representing both rural/town and suburban/city areas) giving a total sample size of 900–1,500 adolescents.

#### 3.4.3 Data gathering

The data gathering consists of a baseline and a one-year follow-up. For the baseline measurement, pupils will be provided with a link to the online questionnaire and given 30 min to fill it out during regular school hours after agreement with each school. The questionnaire will however be made available for 1 month to assure absent pupils and those who prefer to answer it from their home have an opportunity to do so. For the one-year follow-up, pupils are then contacted via e-mail and provided with a link to the same online questionnaire.

The following instruments will be filled in by the participants at the two time points, i.e., at baseline and 1 year follow-up:

- The Social Capital Questionnaire for adolescents that has been developed in WP 1.
- The adapted version of Cantril’s ladder is a well-recognized and validated instrument to assess life satisfaction (56). It has been a mandatory part of the Health Behaviour in School-aged Children survey for 20 years and uses a ladder-type response, where the informant is asked to rate their satisfaction with life from a scale of 0–10 (0 implies worst possible life and 10 best possible life) (56).
- Health Behaviour in School-aged Children Symptom Checklist (HBSC-SCL) is also a well-recognized and validated instrument that comprises eight mental health problems that are indicative of a psychosomatic strain on the body (57). Also included in the HBSC survey, the informant is asked to rate how often during the past 6 months they have experienced each health complaint (headache, abdominal pain, back pain, dizziness, bad temper, trouble sleeping, feeling low, feeling nervous) and response options range between 1 (never) to 5 (almost every day).
- The Mental Health Continuum (MHC-SF) is a self-report measure increasingly used to measure positive mental health and psychological wellbeing in various populations (58, 59). The MHC-SF includes both aspects of wellbeing, hedonia (emotional wellbeing and happiness), and eudaimonia (social function, wellbeing, and social relations). It includes 14 questions and has been shown to be a psychometrically sound instrument for measuring mental health wellbeing in a general population of Swedish adolescents (60).
- The Help-seeking questionnaire for adolescents that has been developed in WP1.

Additional background characteristics will concern; gender (boy/girl/other gender identity), country of birth, parents’ country of birth, extended family abroad, subjective economic situation, housing situation.

#### 3.4.4 Statistical analysis

The quantitative data will be analysed using the statistical computer software SPSS v.28 (IBM) and Mplus (Muthén & Muthén) to allow for descriptive investigation of the data, correlation and multilinear regression analyses, and Latent Profile Analysis (LPA). LPA is used to uncover latent patterns in data sets using variables that are more or less correlated with each other (61). This sort of analysis is favourably used to avoid setting arbitrary threshold-values and to generate an understanding that goes beyond investigations of simple linear associations. This is particularly suitable for the detection of patterns in data where complex interactions between multiple factors occur. Such as in between social capital, mental health and help-seeking.

### 3.5 WP 3 – co-production

The main research question addressed in the co-production WP is: How can the knowledge generated throughout the project be translated into practice? This WP runs parallel with WP 1 and 2. It’s designed to evolve the generated knowledge and maximize its’ applicability during, rather than after, the project is finalized, thus, a process-oriented method will be applied. We do not wish to produce an academic product or program that is “dropped” on various bodies, such as schools or the student-health workers, but rather integrate potential users’ and stakeholders’ perspectives and knowledge to create something that will be viable, sustainable and useful from their perspectives. The idea of the participatory research process is to work towards a common goal, which is to improve early identification of adolescents at risk of developing severe mental health problems.

#### 3.5.1 Participants and recruitment

All pupils participating in WP 1 are offered to join WP 3 in the role of experts representing the target group. Stakeholder perspectives will involve teachers and school health services, social services or strategic positions in each municipality and representatives from organizations that work with adolescent mental health promotion. Invitations, general information about the project and specific agenda for each session will be sent out beforehand.

#### 3.5.2 World-Café

In order to reach the common goal, the World Café (WC) method will be employed (62). The WC method makes it possible to involve adolescents and other stakeholders during the research process, thus providing a cross-pollination with a focus on constructive dialogue and practical implications of shared insights during the process. The WC method is an inclusionary method purposefully designed to enable large group discussions, facilitating dialogue and knowledge exchange. Rounds of conversation take place between participants seated at café-style tables. One host remains whereas the other participants move tables and continue the discussions. During discussions notes, sketches and symbols are documented by the participants on the paper tablecloth or similar. Lastly, a whole group discussion takes place where common discoveries and insights can be shared. The WC method contains explicit instructions on how to reduce power inequalities that may exist between participants. Within scientific research there is an ongoing paradigm shift, moving away from top-down approaches and knowledge produced primarily for the scientific community to bottom-up participatory research approaches, not least in research in matters involving children and adolescents (62).

WC workshops are planned continuously during the project. As this is a fluent process, partly designed to fit the needs of participating schools and stakeholders, we will not set a specific count on World Café sessions. We aim to conduct at least three sessions, one after WP 1, one after Baseline in WP 2 and a third after the one-year follow-up. Discussions in the first session will emanate from the themes generated from the focus group interviews in WP 1 and will focus on knowledge-sharing regarding social capital, mental health, and help-seeking. Later, focus will lie on the findings from the initial analysis of the baseline-survey, presenting preliminary latent profiles and their characteristics and how they may be interpreted. The statistical analysis applied on the data from the baseline-survey provides highly probable typologies of adolescents that have differentiating social capital, mental health outcomes and help-seeking patterns, which are suitable for discussion. Towards the end of the project discussions will focus on the findings from the analysis of the follow-up survey. We foresee that these sessions will have a larger format where the preliminary findings will be presented by the research team in addition to the proceeding discussions.

#### 3.5.3 Knowledge translation and collaboration

These occasions serve as an opportunity to reflect on what is achieved, provide a platform for practitioners and adolescents to ask questions and highlight and discuss the practical implications of the research findings on all levels, from adolescents to stakeholders to researcher. One strength of WC is the before-mentioned cross- pollination of ideas through information exchange and the use of a café-style social context that facilitates the sharing of information in a friendly and equitable environment (62). The advantages of the WC method are multiple. First, it offers a relaxed environment where diverse groups can share different views of a collective phenomenon. Second, it encourages a constructive dialogue, with mutual and reflective learning among participants that well supports the development of new ideas. Third, the sessions enable the possibility of collective discoveries that move participants beyond information transfer to information exchange. Lastly, researchers observe the discussions and have a free role, moving between session groups to help facilitate discussions without taking on a moderating role (62). The research team will collect, compile, and disseminate the output from each session to ensure that the participants are given an opportunity to review and reflect on the output.

#### 3.5.4 Identifying the next steps

The research team will be open to have the output of the WC sessions inform the continuation of the project, by considering new ideas and exploration of new variables within the boundaries of the existing design. Furthermore, the research team will function as a partner onwards to assist stakeholders in translating the research findings and help to identify the next steps of implementing the knowledge generated throughout the project into practice.

### 3.6 Ethics statement

The project has received ethical approval from the Swedish Ethical Review Agency (Ref nr: 2023-01531-01). The studies will adhere to the principles of research ethics according to The Declaration of Helsinki (46), underscoring the importance of informed consent and voluntariness. All participants will receive oral and written information about the study aims and procedures and will be required to provide their written informed consent to participate prior to participation.

## 4 Discussion

### 4.1 Expected results

This project addresses the concerning public health challenge of increased mental health problems among adolescents and the paradoxical development of healthcare utilization and help-seeking (9–11). Converging evidence highlights the critical role of the social environment in influencing adolescent mental health (63). Therefore, there is great potential in exploring further the relationship between social capital and mental health, considering and building on previous research (12, 16, 23, 64). This project adds the scope of help-seeking, for improvement of preventive and promotive mental health work.

- The main outcome of WP 1 will be a relevant and up-to-date conceptualisation of the underpinning constructs of the concept of social capital based on the interactions with Swedish adolescents, and validated instruments to assess both social capital and help-seeking, functioning as a prerequisite for continued investigation.
- Expected outcome of WP 2, is a furthered understanding of the relationship between adolescents’ social capital, mental health and help-seeking, facilitating early detection of adolescents at risk of developing severe mental health problems. We expect to gain insights from the latent profile analysis into how patterns and changes in social capital and mental health are linked to help-seeking, both pertaining to informal sources and mental health care providers. This will not involve determination of certain threshold values but instead provide meaningful means of identifying and communicating vulnerability in adolescence. It is our determination to contribute to a deeper understanding of the mechanisms behind the paradoxical help-seeking patterns among adolescents today (10, 11). Developing state of the art instruments will help explore the complex associations between social capital, mental health and help-seeking with greater accuracy and validity than before. We will hopefully disentangle some of the complexity in these relationships and bring some clarity to the role of social capital as a key component of adolescent mental health promotion. Moreover, the longitudinal design will allow for investigation of how changes in social capital influence mental health and help-seeking behaviour and intentions. This will make a crucial contribution to the question of causality between social capital, mental health and help-seeking for adolescents. Longitudinal research in the adult population has already shown the importance of integrating social and civic participation in general and mental health promotion and support (65), and our intention is to add further to this research. Additionally, by distinguishing between different adolescent groups, we will also be able to discern patterns of social capital, thus rendering a better possibility of tailoring interventions according to specific needs. An aspiration is to prolong this project to follow the adolescents into adulthood, to create conditions for deeper insights.
- WP 3 holds considerable promise to set a precedent of how to narrow the gap between research and practice to produce sustainable and efficient strategies to facilitate help-seeking and improve the mental health of adolescents within existing organizational structures.

### 4.2 Strengths and limitations

A limitation lies in the recruitment of schools willing to participate in the project. The approach as it is designed, may be perceived as burdensome by schools that face challenges with for example staffing, absenteeism, student violence, and poor student-teacher relationships. This may affect the representativeness of the school sample but is a natural consequence of voluntariness in research. The previously established contacts with municipal stakeholders involve schools from different socioeconomic settings, which will help compensate for this limitation. A strength of this project is the participatory approach, which values the knowledge and experiences of the target group and relevant stakeholders to help bridge the gap between research and practice. There are always challenges related to participation when involving young people in research (66). These relate to attaining a level of participation that encompasses authentic opportunities for participants to exert influence and structuring this engagement in a manner that fosters their comprehension of the nature and rationale behind their involvement. The key to overcoming this challenge lies in facilitating sustained participation throughout the project, allowing adolescents to gain confidence in the researchers and help improve their decision-making capabilities (41). One possible limitation is the finite transferability of the instruments developed in this project. It concerns validity over different social cultures and contexts, regions, or countries, affected by societal structures and resources. Similarly, future technical advancements relating to help-seeking may infer temporal sensitivity in unanticipated ways. Nonetheless, there will invariably be challenges relating to sustainability in the development of instruments aimed at capturing social phenomena. Another aspect of transferability concerns representativeness of the sample. Researchers should evidently be aware of the setting that their research is being conducted in, and what groups are represented in the sample. In our case, characteristics of participating schools, diversity of pupils etc., are considered carefully since these features will guide inferences and implications of the results. Lastly, we want to point out that the focus of this project is not on a particular vulnerable group, such as adolescents diagnosed with mental illness, but on adolescents in general within the societal context where this project takes place. We are, however, aware of the possibility of encountering vulnerable individuals during the project and will use the experience within the research team to guide individuals to appropriate care.

## Ethics statement

The project has received ethical approval from the Swedish Ethical Review Agency (Ref nr: 2023-01531-01). The studies will adhere to the principles of research ethics according to The Declaration of Helsinki (46), underscoring the importance of informed consent and voluntariness. All participants will receive oral and written information about the study aims and procedures and will be required to provide their written informed consent to participate prior to participation.

## Author contributions

MA: Conceptualization, Funding acquisition, Methodology, Writing – original draft, Writing – review & editing. AM: Conceptualization, Writing – review & editing. PS: Conceptualization, Writing – review & editing. JN: Conceptualization, Writing – review & editing. ME: Conceptualization, Methodology, Writing – review & editing. KW: Conceptualization, Funding acquisition, Methodology, Writing – original draft, Writing – review & editing.
